# On the viability of *Escherichia coli *cells lacking DNA topoisomerase I

**DOI:** 10.1186/1471-2180-12-26

**Published:** 2012-02-28

**Authors:** Anna Stockum, Robert G Lloyd, Christian J Rudolph

**Affiliations:** 1 Centre for Genetics and Genomics, University of Nottingham, Queen's Medical Centre, Nottingham, NG7 2UH, UK; 2 Division of Medicine, Imperial College London, St Mary's Campus, Norfolk Place, London, W2 1PG, UK; 3 Division of Biosciences, School of Health Sciences and Social Care, Brunel University London, Uxbridge, UB8 3PH, UK

## Abstract

**Background:**

Manipulations of the DNA double helix during replication, transcription and other nucleic acid processing cause a change of DNA topology, which results in torsional stress. This stress is relaxed by DNA topoisomerases, a class of enzymes present in all domains of life. Negatively supercoiled DNA is relaxed by type IA topoisomerases that are widespread in bacteria, archaea and eukaryotes. In *Escherichia coli *there is conflicting data about viability of *ΔtopA *cells lacking topoisomerase I.

**Results:**

In this study we sought to clarify whether *E. coli *cells lacking topoisomerase I are viable by using a plasmid-based lethality assay that allowed us to investigate the phenotype of *ΔtopA *cells without the presence of any compensatory mutations. Our results show that cells lacking topoisomerase I show an extreme growth defect and cannot be cultured without the accumulation of compensatory mutations. This growth defect can be partially suppressed by overexpression of topoisomerase III, the other type IA topoisomerase in *E. coli*, suggesting that the accumulation of torsional stress is, at least partially, responsible for the lethality of *ΔtopA *cells. The absence of RNase HI strongly exacerbates the phenotype of cells lacking topoisomerase I, which supports the idea that the processing of RNA:DNA hybrids is vitally important in *ΔtopA *cells. However, we did not observe suppression of the *ΔtopA *phenotype by increasing the level of R-loop processing enzymes, such as RNase HI or RecG.

**Conclusions:**

Our data show unambiguously that *E. coli *cells are not viable in the absence of DNA topoisomerase I without the presence of compensatory mutations. Furthermore, our data suggest that the accumulation of R-loops is not the primary reason for the severe growth defect of cells lacking topoisomerase I, which is in contrast to the current literature. Potential reasons for this discrepancy are discussed.

## Background

Cellular growth and division requires unwinding of millions of base pairs to allow duplication of chromosomes or to produce the RNA transcripts needed to express genes. Unwinding of the double helix results in torsional stress, a stress solved by topoisomerases, a ubiquitous group of enzymes that are capable of managing the topological state of DNA. Topoisomerases transiently break either one (type I topoisomerases) or both strands (type II topoisomerases) of the double helix, change the topological state of the DNA and then re-ligate the break. This manipulation enables not only modification of DNA superhelicity to allow unwinding of the double helix, but allows the decatenation of circular DNAs, thereby enabling circular chromosomes or plasmids to be separated during cell division [1-3].

In *Escherichia coli *one of the best studied examples of a type IA topoisomerase (where the protein link is to the 5' phosphate, in contrast to type IB topoisomerases where the protein link is to the 3' phosphate) is DNA topoisomerase I, which is encoded by the *topA *gene. Topoisomerase I relaxes negative torsional stress and is required to prevent the chromosomal DNA from becoming extensively negatively supercoiled [4]. Topoisomerase I requires an exposed single stranded region [4]. In *E. coli *the chromosomal DNA is normally slightly negatively supercoiled due to the activity of DNA gyrase, a type IIA topoisomerase, and extensive single stranded regions are not available for topoisomerase I to act on [3]. However, the unwinding of the double helix will result not only in single stranded regions but also in extensive changes in the local level of torsional stress. For instance, the "twin-domain" model of transcription suggests that the elongating RNA polymerase complex (RNAP) causes accumulation of positive torsional stress in front of the transcription complex, whereas negative supercoils accumulate behind [5]. While the positive supercoils are relaxed by gyrase, the negative torsional stress leads to the formation of single stranded DNA, which is a hot-spot for relaxation by topoisomerase I [4].

In cells lacking the activity of topoisomerase I the chromosomal DNA becomes hypernegatively supercoiled, especially behind transcribing RNAP complexes. DNA gyrase will remove the positive torsional stress in front of RNAP, whereas the negative supercoils will persist if they cannot be relaxed by Topo I. This accumulation of negative supercoils has been thought to increase the probability that the newly generated transcript will hybridise with the template strand, thereby forming an R-loop [6]. This idea was supported by results showing that R-loops are a substrate for topoisomerase I in vitro [4]. Furthermore, increased levels of RNase HI, encoded by the *rnhA *gene, have been shown to partially suppress the growth defect of *ΔtopA *cells, while the deletion of *rnhA *exacerbated the *ΔtopA *phenotype [7].

It was initially described that *ΔtopA *cells can grow without apparent ill effect [8]. However, it was later discovered that the *ΔtopA *mutant strains used had accumulated compensatory mutations in DNA gyrase and that *ΔtopA *strains without these suppressor mutations show a severe growth defect [9], an observation confirmed in later studies [7]. It is not clear why growth of cells lacking topoisomerase I is so severely impeded. The genetic interaction of *topA *and *rnhA *has led to the suggestion that R-loops might be a major problem for *ΔtopA *cells. However, *ΔtopA *strains have been reported to be viable in *Salmonella *[10], a result that prompted Stupina and Wang to re-investigate the viability of *E. coli *cells lacking topoisomerase I and they reported that viable *ΔtopA *derivatives can indeed be engineered [11].

In this study we employed a plasmid-based lethality assay [12,13] to investigate the viability and the phenotypes of *ΔtopA *cells without the presence of any compensatory mutations. Our data show that cells lacking topoisomerase I suffer from an extreme growth defect and cannot be subcultured unless they acquire compensatory mutations. This growth defect was suppressed by overexpression of topoisomerase III, the other *E. coli *type IA topoisomerase, as reported [4,14]. We show that deletion of *rnhA *strongly exacerbates the phenotype of cells lacking Topo I, which supports the idea that processing RNA:DNA hybrids is vitally important in the absence of topoisomerase I. However, in contrast to previous results [7] we did not observe any suppression of the *ΔtopA *phenotype if the level of R-loop processing enzymes (RNase HI, RecG) was increased, suggesting that R-loops are not the primary reason for the lethality of *ΔtopA *single mutants.

## Results and discussion

To investigate whether a *ΔtopA *strain can grow without compensatory mutations we employed a plasmid-based lethality assay [12,13]. The wild type *topA *gene was cloned into pRC7 (pAST111), a *lac^+ ^*mini-F plasmid that is rapidly lost from cells. This was used to compensate for a *topA::apra *null mutation in the chromosome of a *Δlac *background. If a *ΔtopA *mutant is viable, plasmid-free cells will form white *lac^- ^*colonies on agar plates supplemented with X-gal and IPTG. However, if a *topA *deletion is lethal, cells that have lost the plasmid will fail to grow, allowing only formation of blue *lac^+ ^*colonies. When viability is reduced but not eliminated, the colonies formed by cells retaining the plasmid are noticeably larger than those formed by plasmid-free cells [13,15].

**Figure 1:**
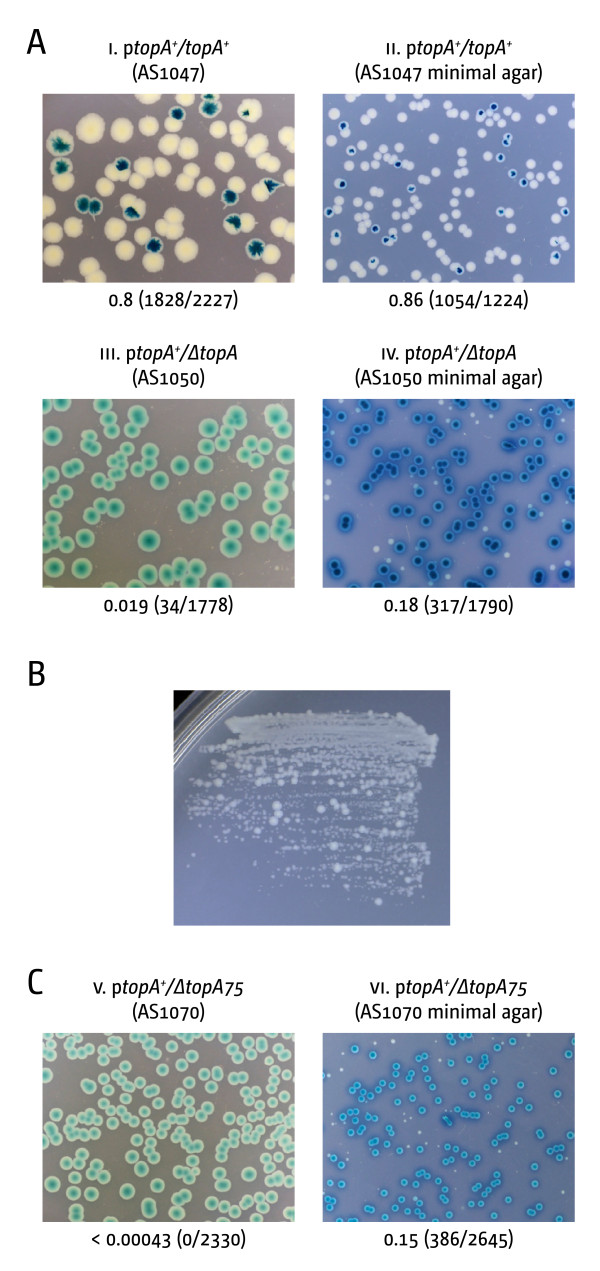
**Maintenance of cell viability in the absence of DNA topoisomerase I**. **(A)** Effect of the deletion of *topA*. The plate photographs shown are of synthetic lethality assays. These, and similar assays reported in subsequent Figures, are described in detail in Materials and Methods. The relevant genotype of the construct used is shown above each photograph, with the strain number in parentheses. The fraction of white colonies is shown below with the number of white colonies/total colonies analyzed in parentheses. **(B) **Abortive growth of a plasmid-free colony from panel A iv after re-streaking on minimal medium. Large colony variants indicate the rapid accumulation of suppressor mutations in a *topA *single mutant. **(C)** Effect of *ΔtopA75 *on viability

As shown by the absence of large plasmid-free (*lac^-^*) colonies (Figure 1A), *ΔtopA::apra *cells without topoisomerase I are extremely sick on LB agar. This severe phenotype was only little affected by different temperatures or salt concentrations (Additional file 1: Figure S1A and Additional file 1 S1B) [11,16,17]. On minimal medium, white colonies were observed (Figure 1A, panel iv) but they rapidly accumulated suppressor mutations upon re-streaking onto minimal medium (Figure 1B). We repeated the experiment using the *ΔtopA75 *allele used in the study of Stupina and Wang [11], which gave identical results (Figure 1C, panel v and vi). Thus, our assay shows that cells lacking DNA topoisomerase I are extremely sick and grow very slowly, but develop suppressors, suggesting that the selective pressure for compensatory mutations such as mutations in *gyrA*, *gyrB*, *tolC *and *topB *[4,14,18] is very high.

### The Δ*topA *lethality is suppressed by overexpression of *topB*

Many of the studies investigating the properties of *ΔtopA *cells have worked in a background with a conditional *gyrB *mutation. Mutations in *gyrA *or *gyrB *reduce the global level of supercoiling, thereby enabling *ΔtopA *cells to grow [4]. In *gyrB203*(ts) strains the activity of gyrase is reduced at high temperature. Thus, *ΔtopA gyrB203(ts) *cells grow at high temperature, since the reduced activity of gyrase compensates of the absence of topoisomerase I, but are cold-sensitive [4]. By using the plasmid-based lethality assay we were able to investigate some of the properties of *ΔtopA *cells without the presence of a compensatory mutation.

We repeated overexpression studies with *topB*, which encodes for topoisomerase III, the other member of the type IA family of topoisomerases in *E. coli *[4]. DNA topoisomerase III was shown to relax transcription-induced negative supercoiling in vivo and in vitro [4] and high levels of expression partially suppressed the growth defect of *ΔtopA *strains [14]. To investigate the effect of *topB *overexpression in a *topA *deletion background we used pECR17, a P*_araBAD _topB *expression plasmid that allows arabinose-controlled expression of *topB*. For these experiments cultures were grown overnight, with selection for both pECR17 and pRC7 *topA*. The cultures were then diluted as described in Material and Methods and parallel cultures grown with the arabinose concentration indicated, selecting only for pECR17. The cultures were then diluted as described and plated on plates with the corresponding arabinose concentration and selection for pECR17.

**Figure 2:**
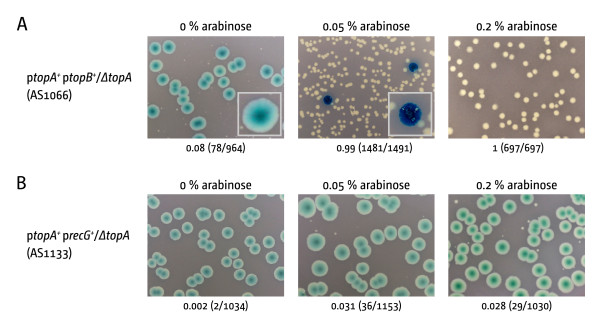
**The lethality of *ΔtopA *cells can be suppressed by increased levels of DNA topoisomerase III, but not by overexpression of *recG***. **(A) **Arabinose-induced expression of *topB*, which codes for DNA topoisomerase III, leads to formation of white colonies. The smaller colony size indicates that the suppression is only partial. Increased levels of DNA topoisomerase III are toxic for *E. coli *cells, leading to reduced numbers of blue colonies as well as aberrant colony morphologies (compare the two enlarged colonies in Ai and Aii). **(B) **Increased levels of RecG support growth of *ΔtopA *cells only marginally

Formation of white colonies was observed if expression from the P*_araBAD _*promoter was induced with medium and high levels of arabinose, confirming that *topB *is a multicopy suppressor of *ΔtopA *(Figure 2A). The white colonies were smaller in size, suggesting that overexpression of *topB *suppressed the phenotype of *topA *cells only partially, as observed before [14]. We also observed a strong decrease in the number of blue colonies with increasing levels of *topB *expression and the blue colonies showed a rather unusual morphology (enlarged colonies are shown as insets in Figure 2A panel i and ii). It appears that the overexpression of *topB *prevents growth of cells that retain the *topA *plasmid, in line with previous results showing that increased levels of topoisomerase III are toxic for *E. coli *wild type cells [14,19].

### The Δ*topA *lethality is not suppressed by overexpression of *rnhA *or *recG*

It was previously reported that the growth defect of cells lacking topoisomerase I can be suppressed by increased concentrations of RNase HI. Furthermore, *ΔtopA ΔrnhA *double mutants were found to be inviable even in the presence of point mutations that strongly suppress the *ΔtopA *phenotype [7]. This led to the suggestion that RNA:DNA hybrids might be a major problem for *ΔtopA *cells [7]. We therefore investigated whether RecG helicase suppressed the *ΔtopA *phenotype. RecG protein was shown to unwind the RNA from R-loops in vitro [20,21] and overexpression of *recG *results in reduced yields of ColEI plasmids that initiate replication via an R-loop [20], suggesting that RecG can process R-loops in vivo.

To investigate whether *recG *overexpression suppresses the *ΔtopA *phenotype we used an overexpression construct as described for *topB *(see Material and Methods). The plasmid fully suppressed the phenotype of cells lacking RecG if expression was induced, whereas no suppression was observed under conditions where expression was repressed [22]. As shown in Figure 2B expression of *recG *at high levels only marginally suppressed the *topA *phenotype.

**Figure 3:**
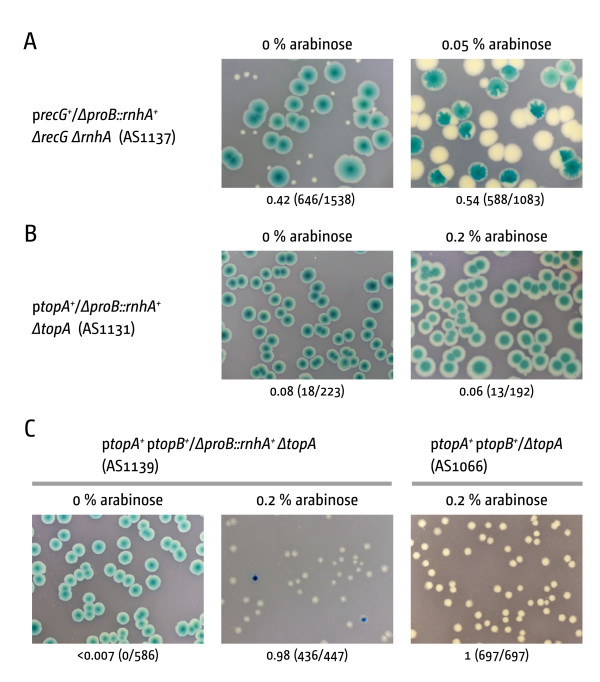
**The lethality of *ΔtopA *cells is not suppressed by increased levels of RNase HI**. **(A) **Expression of a P*_araBAD _rnhA *construct integrated into the chromosome can be regulated by different arabinose concentrations. The expression level is high enough to suppress the synthetic lethality of *rnhA recG *cells. **(B) **Expression from the integrated P*_araBAD _rnhA *construct does not suppress the lethality of *ΔtopA *cells. The P*_araBAD _rnhA *construct has been integrated into a *rnhA^+ ^*background. Thus, expression of the construct will produce RNase HI in addition to the regular *rnhA *locus. **(C) **Expression from the integrated P*_araBAD _rnhA *construct does not improve growth of cells in which the *ΔtopA *defect is partially suppressed by overexpression of DNA topoisomerase III. The image for AS1066 was reproduced from Figure 2 for comparison. Please note that incubation and image capturing procedures are standardised to allow comparison of colony sizes

Our data suggest that R-loop processing activity of RecG is not sufficient to suppress the *ΔtopA *phenotype efficiently. To confirm that elevated concentrations of RNase HI suppress the growth defect of cells lacking topoisomerase I we repeated the experiment with a P*_araBAD _rnhA *plasmid. However, medium expression levels of *rnhA *from a P*_araBAD _*plasmid proved toxic for the cells (Additional file 2: Figure S2), presumably because the high levels of RNase HI degrade the R-loop required to initiate replication at the pMB1 origin. To avoid this problem we amplified the *rnhA *locus including the arabinose promoter region and integrated the construct into the *proB *locus, using standard single-step gene replacement [23]. To confirm that expression of the chromosomal *rnhA *construct can be controlled by arabinose we tested whether it could suppress the synthetic lethality of *rnhA recG *cells [15,24]. We generated a *rnhA recG proB::rnhA^+ ^*strain in which the *recG *deletion was covered by pJJ100 (pRC7 *recG^+^*). As shown in Figure 3A, only very small white colonies were observed after incubation for 48 h on LB agar without arabinose. These white colonies are formed due to the leakiness of the *araBAD *promoter. In contrast, on LB agar with moderate arabinose concentrations robust segregation of blue and white colonies was observed, with the white colonies being as healthy as the blue. Thus, expression of the integrated *rnhA *construct can be regulated by the presence or absence of arabinose.

To test whether increased levels of RNase HI can suppress the lethality of *topA *strains we integrated our *proB::rnhA^+ ^*expression construct into an *rnhA^+ ^*background. Thus, any expression from our integration construct will be in addition to the expression from the native *rnhA *gene. We then introduced our *topA::apra *allele, covering the deletion with the pRC7 *topA *plasmid. However, growth of this strain in medium with moderate (data not shown) or high arabinose concentrations did not lead to formation of white colonies (Figure 3B).

Since we did not directly measure the concentration of RNase HI in cells we cannot exclude the possibility that the levels in our expression constructs are not high enough for suppression of the *ΔtopA *phenotype. We therefore wanted to test the expression of *rnhA *in a system that might be more sensitive for low expression levels. It was observed before that the co-expression of both *rnhA *and *topB *resulted in a synergistic suppression of the *topA *phenotype [14]. We therefore wanted to know whether the expression of *rnhA *from our integration construct would increase the suppression of the observed *topB *overexpression. To test this we transformed our p*topA/ΔtopA ΔproB::rnhA^+ ^*background with the *topB *expression plasmid. However, co-expression did not lead to an increase in the size of the white colonies. If anything a mild reduction of viability is observed (Figure 3C).

### Absence of RNase HI strongly exacerbates the phenotype of *ΔtopA *cells

**Figure 4:**
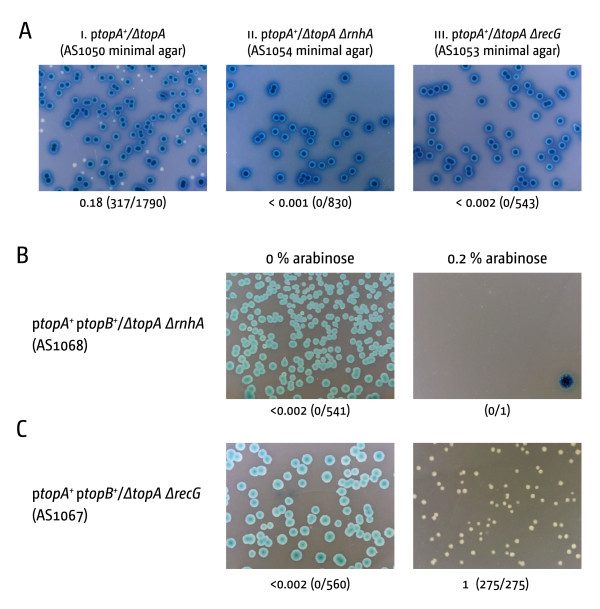
***rnhA *and *recG *deletions exacerbate the *ΔtopA *phenotype**. **(A) **No white colonies are observed on minimal medium when *ΔtopA *is combined with either *rnhA *or *recG*. **(B-C) **Overexpression of *topB *in a p*topA^+^*/*topA recG *background allows formation of white colonies. The overexpression of *topB *in p*topA^+^*/*topA rnhA *cells has no effect

The data presented imply that increasing levels of RNA:DNA processing enzymes appears not to be enough to suppress the phenotype of cells lacking topoisomerase I. Since we used an experimental system that was independent of *ΔtopA *compensatory mutations there might be a number of reasons for the observed differences. The available *topB *overexpression data suggest that *ΔtopA *cells suffer from strong topological defects. It is possible the *gyrB203(ts) *compensatory mutation alleviated some of these defects even at low temperature, which might enable increased levels of RNase HI to suppress the phenotype even further [14]. Alternatively, the level of RNA:DNA hybrids might be very high. Since we did not measure the expression level of our *ΔproB::rnhA^+ ^*directly, we cannot exclude the possibility that the *rnhA *expression level is not high enough for suppression of the *ΔtopA *phenotype. To investigate whether an RNA:DNA hybrid processing activity is important in the absence of Topo I we generated a *ΔrnhA ΔtopA *double mutant, as it was described before that *topA rnhA *double mutants are inviable even if *topA *is suppressed by strong suppressor mutations such as *gyrB203(ts) *[7]. We noticed that *ΔrnhA ΔtopA *double mutants were not able to form white colonies on minimal medium, which suggests that the deletion of *rnhA *indeed exacerbates the *topA *phenotype (Figure 4A panel ii). We transformed the p*topA/ΔtopA ΔrnhA *strain with our P*_araBAD _topB *overexpression plasmid to verify that the *ΔrnhA ΔtopA *double mutant can be partially suppressed by overexpression of *topB*, as reported [25]. However, overexpression of *topB *did not suppress the synthetic lethality of *ΔrnhA ΔtopA *cells in our system (Figure 4B). Cells cannot grow in the absence of the *topA *plasmid despite the overexpression of *topB*. However, in cells retaining the topA plasmid the high levels of topoisomerase III is toxic, which explains the almost total absence of colonies (Figure 4B). Thus, the resolution of topological stress does not render *ΔtopA *viable if the major enzyme that processes DNA:RNA hybrids is absent.

Our results show that RecG can not compensate for the absence of RNase HI (Figure 2). However, if RecG processes some R-loops in vivo, the deletion of *recG *in a *ΔtopA *background should exacerbate the *topA *phenotype, as observed with *rnhA*. This was indeed observed. *ΔrecG ΔtopA *double mutants were not able to form any white colonies, neither on LB broth (data not shown) nor on minimal medium (Figure 4A panel iii). However, when we transformed the construct with the P*_araBAD _topB *overexpression plasmid we observed that overexpression of *topB *allowed growth of *ΔrecG ΔtopA *cells, with white colonies being only marginally smaller than in the *topA*/p*topB^+ ^*control (compare Figures 4C and Figure 2A). Thus, the impact of a *recG *deletion is marginal in comparison to the impact of deleting *rnhA*, suggesting that the contribution of RecG to genome-wide processing of R-loops might be lower than anticipated.

## Conclusion

The plasmid-based lethality assay exploited in this study provided a novel approach to investigate the phenotype of cells lacking topoisomerase I without the presence of any compensatory mutations. The results presented show that cells lacking topoisomerase I exhibit an extreme growth defect, indicating that they are under a constant selection pressure for compensatory mutations. This phenotype was partially suppressed by overexpression of topoisomerase III, suggesting that the accumulation of torsional stress is, to a certain extent, responsible for the lethality of *ΔtopA *cells, as reported [14]. However, the overexpression of R-loop processing enzymes, such as RNase HI or RecG, did not result in a major suppression of the *ΔtopA *phenotype. This result suggests that the accumulation of R-loops does not contribute very much towards the growth defect of cells lacking topoisomerase I, which is in contrast to previous reports [4,7]. However, the absence of RecG and especially RNase HI exacerbates the phenotype of *ΔtopA *cells, which suggests that the processing of RNA:DNA hybrids is vitally important in the absence of topoisomerase I. Thus, R-loops accumulate to a toxic level only in cells lacking RNase HI, while the toxicity in *ΔtopA *single mutants is mainly caused by an additional effect that is yet to be characterised. Further experiments will be necessary to shed light on the question as to why cells lacking Topo I have such a severe growth defect and how much R-loops contribute to this phenotype.

## Methods

### Strains

Bacterial strains are listed in Table 1. All constructs used for synthetic lethality assays are based on *E. coli *K-12 MG1655 *ΔlacIZYA *strains carrying derivatives of pRC7 (Bernhardt and de Boer 2004). The deletion allele of *topA *(*ΔtopA::apra*) was made using the one-step gene disruption method of Datsenko and Wanner [23]. The *ΔtopA::apra *allele removes all but 45 bp from the 5' and 3' end of the coding sequence. The *proB::*P*_araBAD _rnhA *was generated by standard single-step gene replacement [23]. pECR15 was cleaved with *Hin*dIII and the *Hin*dIII *frt-kan-frt *cassette from pDIM141 (see below) ligated into the construct. The resulting plasmid was used for amplification of P*_araBAD _rnhA frt-kan *with the primers introducing 40 bp of sequence homologous to *proB*. The construct was integrated into the *proB *locus and the kanamycin resistance marker removed via FLP recombinase [23].

**Table 1:** *Escherichia coli *K-12 strains

Strain	Relevant genotype	Source
**General P1 donors**		

VS111	F^- ^*ΔtopA75 zci2234::cat Δfnr-267? rph-1*	CGSC [11]

**MG1655 and derivatives**		

N4560	*ΔrecG::cat*	[26]

N4704	*rnhA::cat*	[15]

N6052	*ΔrecG::apra*	[13]

AM2283	*ΔlacIZYA ΔproB::rnhA^+^-frt > kan > frt*	This study

AM2284	*ΔlacIZYA ΔproB::rnhA^+^- frt > kan > frt *pCP20	AM2283 × pCP20 to Ap^r^

AM2285	*ΔlacIZYA ΔproB::rnhA^+^- frt*	AM2284

AM2290	*ΔlacIZYA ΔproB::rnhA^+^- frt > kan > frt*	TB28 × P1.AM2283 to Km^r^

AM2304	*ΔlacIZYA ΔproB::rnhA^+^- frt > kan > frt ΔrecG::apra*	AM2290 × P1.N6052 to Apra^r^

AS1047	*ΔlacIZYA *pAST111	TB28 × pAST111 to Ap^r^

AS1050	*ΔlacIZYA *Δ*topA::apra *pAST111	AS1047 × P1.RCe296 to Apra^r^

AS1053	*ΔlacIZYA topA::apra ΔrecG::cat *pAST111	AS1050 × P1.N4560 to Cm^r^

AS1054	*ΔlacIZYA topA::apra rnhA::cat *pAST111	AS1050 × P1.N4704 to Cm^r^

AS1066	*ΔlacIZYA topA::apra *pAST111 pECR17	AS1050 × pECR17 to Ap^r ^Km^r^

AS1067	*ΔlacIZYA topA::apra ΔrecG::cat *pAST111 pECR17	AS1053 × pECR17 to Ap^r ^Km^r^

AS1068	*ΔlacIZYA topA::apra rnhA::cat *pAST111 pECR17	AS1054 × pECR17 to Ap^r ^Km^r^

AS1070	*ΔlacIZYA ΔtopA75 zci-2234::cat *pAST111	AS1047 × P1.VS111 to Cm^r^

AS1130	*ΔlacIZYA ΔproB::rnhA^+^-frt *pAST111	AM2285 × pAST111 to Ap^r^

AS1131	*ΔlacIZYA ΔproB::rnhA^+^-frt topA*::apra pAST111	AS1130 × P1.RCe296 to Apra^r^

AS1133	*ΔlacIZYA topA::apra *pAST111 pAST120	AS1050 × pAST120 to Km^r ^(Ap^r^)

AS1134	*ΔlacIZYA ΔproB::rnhA^+^- frt > kan > frt ΔrecG::apra *pJJ100	AM2304 × pJJ100 to Ap^r^

AS1137	*ΔlacIZYA ΔproB::rnhA^+^- frt > kan > frt ΔrecG::apra rnhA::cat *pJJ100	AS1134 × P1.N4704 to Cm^r^

AS1139	*ΔlacIZYA ΔproB::rnhA^+^- frt topA::apra *pAST111 pECR17	AS1131 × pERC17 to Km^r ^(Ap^r^)

RCe296	*topA::apra*	This study

TB28	*ΔlacIZYA*	[12]

### Plasmids

pRC7 is a low copy-number, mini-F derivative of the *lac*^+ ^construct pFZY1 [12]. pJJ100 (*recG^+^*) and pAST111 (*topA^+^*) are derivatives of pRC7 encoding the wild type genes indicated. The construction of pJJ100 has been described elsewhere [13,15,27]. For generation of pAST111 the *topA *gene was PCR amplified from MG1655 chromosomal DNA. To account for the complex promoter of the *topA *gene [28], 150 bp upstream of the start codon were included. Both the 5' and the 3' primer introduced *Apa*I sites, allowing cloning into the *Apa*I site within the *lacI^q ^*gene of pRC7. pAST120 (*recG*^+^), pECR15 (*rnhA^+^*) and pECR16/17 (*topB^+^*) are all P*_araBAD _*derivatives, which allow arabinose-controlled expression of the genes indicated. For the construction of pAST120 the *Hin*dIII fragment from pDIM141 containing a kanamycin resistance marker flanked by FRT sites was cloned into the single *Hin*dIII site of pDIM104, the construction of which was described elsewhere [22]. This allowed maintenance of the plasmid via kanamycin selection. pECR15 (*rnhA*) was constructed by amplifying the *rnhA *gene from MG1655 chromosomal DNA with the 5' primer introducing a *Eco*RI and the 3' primer introducing a *Xba*I site, allowing cloning into P_*araBAD*_. pECR16 (*topB*) was generated in an analogous way. To allow maintenance of the plasmid via kanamycin the *Hin*dIII fragment from pDIM141 was cloned into the single *Hin*dIII site of pECR16, analogous as described for pAST120. pDIM141 is a derivative of pLau17 [29]. The eCFP gene was replaced with mRFP (pDIM117). The kanamycin resistance gene was PCR amplified from EZ-Tn10 with primers introducing FRT sites either side, followed by *Hin*dIII restriction sites. This *FRT-kan-FRT *cassette was then cloned into the single *Hin*dIII site of pDIM117, resulting in pDIM141.

### Media and general methods

LB broth and 56/2 minimal salts media, and methods for monitoring cell growth and for strain construction by P1*vir*-mediated transduction have been cited [30-32].

### Synthetic lethality assays

The rationale for synthetic lethality assays has been described [12,13]. Essentially, a wild type gene of interest is cloned in pRC7, a *lac^+ ^*mini-F plasmid that is rapidly lost, and used to cover a null mutation in the chromosome, in a *Δlac *background. If the mutant is viable, the plasmid-free cells segregated during culture will form *lac^- ^*colonies on agar plates. If, however, the deletion is lethal, they will fail to grow and only *lac^+ ^*colonies formed by cells retaining the plasmid will be observed. When viability is reduced but not eliminated, the colonies formed by cells retaining the plasmid are noticeably larger than those formed by plasmid-free cells. To record the phenotype, cultures of strains carrying the relevant pRC7 derivatives were grown overnight in LB broth containing ampicillin to maintain plasmid selection, diluted 80-fold in LB broth and grown without ampicillin selection to an A_650 _of 0.4 before spreading dilutions on LB agar or 56/2 glucose minimal salts agar supplemented with X-gal and IPTG. Plates were photographed and scored after 48 h (LB agar) or 72 h (56/2 agar) at 37°C, unless stated otherwise. Plasmid-free cells forming small white colonies were re-streaked to see if they could be subcultured, and the streak plates photographed after incubation at 37°C for 24 h to 48 h (LB agar), or 48 h to 72 h (56/2 glucose salts agar), as indicated.

## Authors' contributions

CJR and RGL designed the experiments. AS carried out the experiments. AS, RGL and CJR wrote the manuscript. All authors read and approved the final version of the manuscript.

## Supplementary Material

Additional file 1 **Figure S1**. Viability of cells lacking DNA topoisomerase I at various temperatures and salt concentrations. **(A) **Effect of an increased temperature on *ΔtopA *cells. The plate photographs shown are of synthetic lethality assays as described in detail in Materials and Methods. The relevant genotype of the construct used is shown above each photograph, with the strain number in parentheses. The growth conditions are shown to the left. The fraction of white colonies is shown below with the number of white colonies/total colonies analyzed in parentheses. **(B) **Effect of various salt concentrations on the viability of cells lacking topoisomerase I. Click here for file

Additional file 2 **Figure S2**. The viability of cells increased levels of RNase HI is reduced. Wild type cells carrying a P*_araBAD _rnhA *expression plasmid (pECR15) show a growth defect that depends on the concentration of arabinose present in the growth medium. Even growth on glucose, which suppresses expression from the P*_araBAD _*promoter, leads to a mild growth defect, presumably due to a combination of the high plasmid copy number and the leakiness of the P*_araBAD _*promoter. Cells carrying a control plasmid (P*_araBAD _*eCFP, pAST110) show no growth restriction. Click here for file
