# Supplementary figures and images for: On the viability of Escherichia coli cells lacking DNA topoisomerase I
Source: BMC Microbiol. 2012 Feb 28;12:26. doi: 10.1186/1471-2180-12-26 (PMC3313902; doi:10.1186/1471-2180-12-26)

A

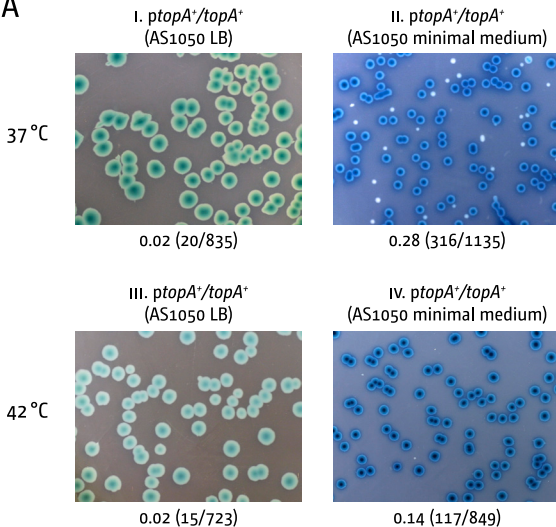

B

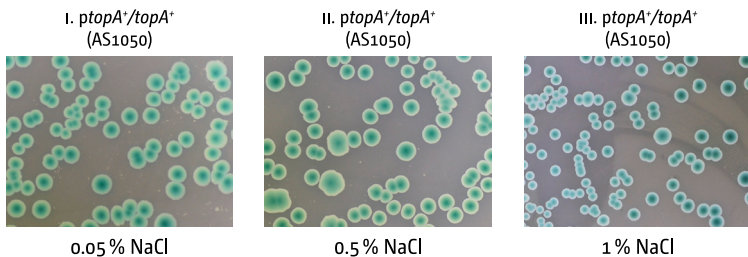

Supplement: Additional file 1 — Figure S1. Viability of cells lacking DNA topoisomerase I at various temperatures and salt concentrations. (A) Effect of an increased temperature on ΔtopA cells. The plate photographs shown are of synthetic lethality assays as described in detail in Materials and Methods. The relevant genotype of the construct used is shown above each photograph, with the strain number in parentheses. The growth conditions are shown to the left. The fraction of white colonies is shown below with the number of white colonies/total colonies analyzed in parentheses. (B) Effect of various salt concentrations on the viability of cells lacking topoisomerase I. [file 1471-2180-12-26-S1.PDF]

PAST110

PCR15

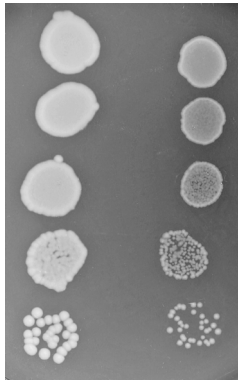

0.2 % glucose

PAST110

PCR15

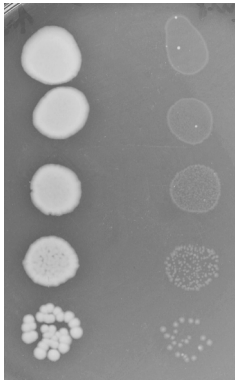

0.05 % arabinose

PAST110

PCR15

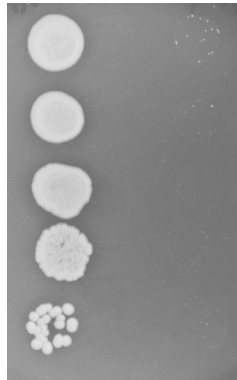

0.2 % arabinose

Supplement: Additional file 2 — Figure S2. The viability of cells increased levels of RNase HI is reduced. Wild type cells carrying a ParaBAD rnhA expression plasmid (pECR15) show a growth defect that depends on the concentration of arabinose present in the growth medium. Even growth on glucose, which suppresses expression from the ParaBAD promoter, leads to a mild growth defect, presumably due to a combination of the high plasmid copy number and the leakiness of the ParaBAD promoter. Cells carrying a control plasmid (ParaBAD eCFP, pAST110) show no growth restriction. [file 1471-2180-12-26-S2.PDF]
